# Shared genetic architecture between periodontal disease and type 2 diabetes: a large scale genome-wide cross-trait analysis

**DOI:** 10.1007/s12020-024-03766-8

**Published:** 2024-03-09

**Authors:** Kevin Chun Hei Wu, Lin Liu, Aimin Xu, Yap Hang Chan, Bernard Man Yung Cheung

**Affiliations:** 1 Department of Medicine, Li Ka Shing Faculty of Medicine, The University of Hong Kong, Pokfulam, Hong Kong SAR, China; 2 State Key Laboratory of Pharmaceutical Biotechnology, The University of Hong Kong, Pokfulam, Hong Kong SAR, China; 3 Division of Cardiology, Queen Mary Hospital, The University of Hong Kong, Pokfulam, Hong Kong SAR, China; 4 Institute of Cardiovascular Science and Medicine, The University of Hong Kong, Pokfulam, Hong Kong SAR, China

**Keywords:** Periodontal Diseases, Glycemic Control, Type 2 Diabetes, Insulin Resistance, Genome Wide Association Analysis

## Abstract

**Purpose:**

To investigate the relationship between abnormal glucose metabolism, type 2 diabetes (T2D), and periodontal disease (PER) independent of Body Mass Index (BMI), we employed a genome-wide cross-trait approach to clarify the association.

**Methods:**

Our study utilized the most extensive genome-wide association studies conducted for populations of European ancestry, including PER, T2D, fasting glucose, fasting insulin, 2-hour glucose after an oral glucose challenge, HOMA-β, HOMA-IR (unadjusted or adjusted for BMI) and HbA1c.

**Results:**

With this approach, we were able to identify pleiotropic loci, establish expression-trait associations, and quantify global and local genetic correlations. There was a significant positive global genetic correlation between T2D (r_g_ = 0.261, *p* = 2.65 × 10^−13^), HbA1c (r_g_ = 0.182, *p* = 4.14 × 10^−6^) and PER, as well as for T2D independent of BMI (r_g_ = 0.158, *p* = 2.34 × 10^−6^). A significant local genetic correlation was also observed between PER and glycemic traits or T2D. We also identified 62 independent pleiotropic loci that impact both PER and glycemic traits, including T2D. Nine significant pathways were identified between the shared genes between T2D, glycemic traits and PER. Genetically liability of HOMA-βadjBMI was causally associated with the risk of PER.

**Conclusion:**

Our research has revealed a genetic link between T2D, glycemic traits, and PER that is influenced by biological pleiotropy. Notably, some of these links are not related to BMI. Our research highlights an underlying link between patients with T2D and PER, regardless of their BMI.

## Introduction

Periodontal disease (PER) refers to a set of inflammatory disorders that affect the tissues that surround the teeth which may lead to the loss of teeth [1]. Type 2 Diabetes (T2D) is a common form of diabetes mellitus characterized by hyperglycemia, impaired insulin sensitivity and insufficient insulin levels. Patients who had severe PER at the beginning of the study were linked to a higher probability of experiencing abnormal blood glucose levels, and vice versa [2, 3].

A two-way relationship was found between T2D and PER in previous study [4]. The impact of PER on individuals with T2D is negative as it affects both the control of blood sugar levels and the development of complications associated with diabetes [5]. Individuals who have T2D are three times more likely to develop PER with greater severity. One of how T2D contributes to the development of PER is through inflammation burden on the periodontium [4]. Accumulating evidence has suggested that obesity increases the risks of periodontal diseases and type 2 diabetes [4, 6], which could potentially act as confounding factors between them. In addition, observational studies also suggest an effect that is independent of BMI [7, 8]. No such analysis has been conducted to comprehensively investigate the relationship between PER and its primary coexisting conditions, abnormal glucose metabolism and T2D considering BMI.

The objectives of this research study were to examine the genetic overlap between T2D, glycemic traits, and PER, with a particular focus on their interaction with or without BMI. A comprehensive genome-wide analysis was carried out using the most extensive genome-wide association study (GWAS) data that was accessible for each of these characteristics. The study focused on individuals of European descent and investigated the impact of T2D and glycemic traits (unadjusted and adjusted for BMI) on the development of PER.

## Methods

### GWAS summary statistics for glycemic traits and Type 2 diabetes

**Fig. 1:**
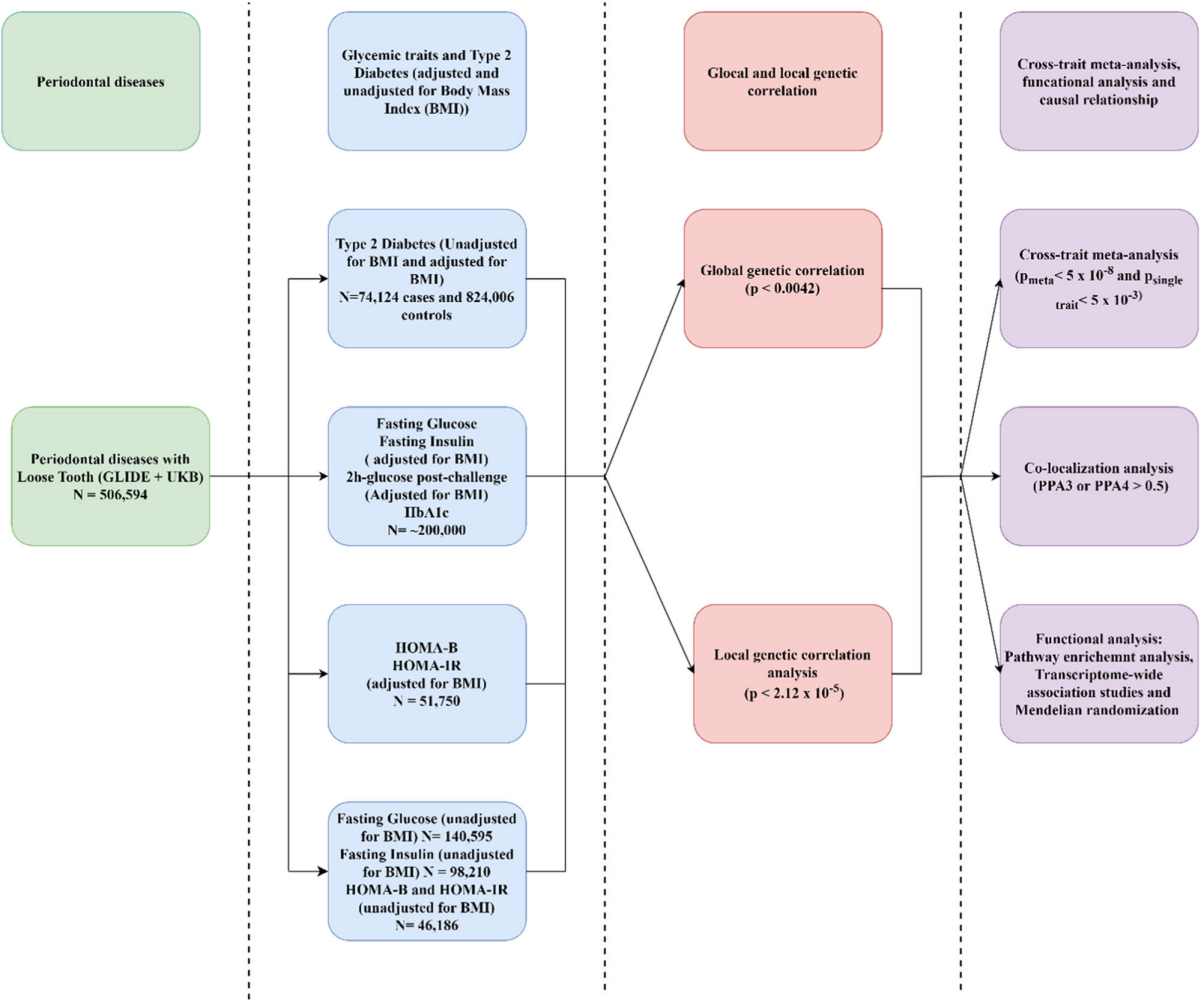
Illustration of the genome-wide cross-trait analysis design. We first quantified global and local genetic correlation then identified specific pleiotropic loci and detected expression–trait associations. Genome-wide global genetic correlation analysis: https://github.com/bulik/ldsc and https://github.com/qlu-lab/GNOVA-2.0 ; local genetic correlation analysis: https://github.com/qlu-lab/SUPERGNOVA; cross-trait meta-analysis: http://hal.case.edu/~xxz10/zhu-web/ ;pairwise analysis: https://github.com/joepickrell/gwas-pw ; transcriptome-wide association analysis: http://gusevlab.org/projects/fusion/ ; pathway enrichment analysis: https://biit.cs.ut.ee/gprofiler/gost ; mendelian randomization: https://mrcieu.github.io/TwoSampleMR/index.html

Figure 1 illustrates the overall study design. We obtained GWAS summary data for T2D and T2DadjBMI from the DIAGRAM consortium, which consisted of 74,124 cases and 824,006 controls [9]. The summary statistics for adjusted BMI glycemic traits (FGadjBMI, FIadjBMI, and GladjBMI) and HbA1c were obtained from the Meta-Analyses of Glucose and Insulin-related traits Consortium (MAGIC), which included around 200,000 European individuals without diabetes [10]. In addition, GWAS summary statistics for the homeostatic model assessment of β-cell function adjusted for BMI (HOMA- βadjBMI) and the homeostatic model assessment of insulin resistance adjusted for BMI (HOMA-IRadjBMI) were also obtained from the MAGIC consortium; the dataset included 58,074 and 51,750 non-diabetic participants in glucose and insulin respectively [11]. The summary statistics for glycemic traits were also obtained from the MAGIC consortium, which included 140,595, 98,210, 46,186, and 46,186 non-diabetic participants for the FG, FI, HOMA-β, and HOMA-IR datasets respectively [12, 13]. We retrieved PER GWAS summary statistics (meta-analysis of periodontitis and loose tooth) obtained from the UK BioBank (UKB) consortium and the Gene-Lifestyle Interactions in Dental Endpoints (GLIDE) [14]. Supplementary Table 1 contains additional information regarding each of the datasets used in the study. The human reference genome build 37 was used in the GWAS summary statistics to perform the analysis.

### Statistical analysis

#### Overall genetic correlation analysis

To evaluate the overall genetic correlation between PER and glycemic traits or Type 2 diabetes, Linkage Disequilibrium Score Regression (LDSC) and Genetic Covariance Analyzer (GNOVA) were employed [15, 16]. Cross trait LDSC was performed by using LD scores from 1000 Genomes Project and the Hapmap3 reference panel with around 1.2 million SNPs [17]. In addition to LDSC, GNOVA was also employed to estimate the genetic correlation between PER and glycemic traits or Type 2 diabetes. In contrast to LDSC, GNOVA utilizes roughly 5 million SNPs from the 1000 Genomes Project, which results in greater statistical power and more accurate estimations. Both LDSC and GNOVA were designed to account for potential sample overlaps between each pair of traits.

#### Local genetic correlation analysis

To pinpoint the precise genomic regions responsible for the genetic associations between the traits, we used SUPERGNOVA to estimate the local genetic correlations between traits in nearly independent LD blocks [18]. In the analysis, 2353 local genomic regions were used. To prevent any potential overlap between samples across different traits, SUPERGNOVA was utilized for control during the analysis. Significance local genetic correlation was based on the threshold of *p* < 2.12 × 10^−5^ (0.05/2353).

#### Cross-trait meta-analysis

To identify shared pleiotropic loci between traits at the level of individual SNPs, cross-trait meta-analysis was performed using cross-phenotype association analysis (CPASSOC) software through the S_het_ statistics [19]. By assigning greater weight to trait-specific effect sizes, the S_het_ statistic is able to maintain its statistical power even in the presence of heterogeneity. Genome-wide significant SNPs were considered if the SNPs reached a threshold of p_CPASSOC_ < 5 × 10^−8^ in CPASSOC and p_single-trait_ < 5 × 10^−3^ between PER and glycemic traits or T2D. Then, independent pleiotropic SNPs were further extracted using the PLINK LD clumping method (parameters: --clump-p1 5e-8 --clump-p2 1e-5 --clump-r2 0.2 --clump-kb 500) [20].

The Ensembl Variant Effect Predictor (VEP) [21] and 3DSNP [22] were used to annotate the independent pleiotropic SNPs between PER and glycemic traits or T2D and find the interacting genes in the independent pleiotropic SNPs.

#### Pairwise colocalization analysis

The GWAS-PW software was used to determine whether there is co-localization between PER and glycemic traits or T2D [23]. GWAS-PW employs a Bayesian framework to compute four different models, each with a posterior probability association. Model 1 indicates that the region contains a genetic variant associated with PER. Model 2 assumes the region contains a genetic variant associated with glycemic traits or T2D. Model 3 suggests that the region has a genetic variant that’s associated with both glycemic traits and PER (PPA3), while model 4 assumes that the region contains two distinct genetic variants, one associated with glycemic traits and the other with PER (PPA4). Regions with PPA3 or PPA4 greater than 0.5 were deemed to have evidence of a shared causal variant or independent causal variants, respectively.

#### Pathway enrichment analysis

The g: profiler tool was used to evaluate the pathway enrichment analysis using the shared loci between PER and glycemic traits or T2D in Gene Ontology (GO) biological processes and Kyoto Encyclopedia of Genes and Genomes (KEGG) pathways [24]. A threshold of FDR < 0.05 are considered as a significant pathway.

#### Transcriptome-wide association studies

The FUSION package was used to perform a transcriptome wide association study in multiple tissues for PER, glycemic traits and T2D. This analysis utilized expression weights derived from 48 post-mortem tissues that were obtained from the GTEx database (version 7) [25]. Then, we compared the significant gene-tissue pairs for each trait to determine if they were shared across different traits. A false discovery rate (FDR < 0.05) was used to account for the multiple testing.

#### Bidirectional Mendelian Randomization (MR) analysis

A bidirectional MR analysis using inverse-variance weighted (IVW) as the primary method was conducted [26]. Sensitivity analyses were performed using Median-based methods (simple, weighted and penalized weighted) [27], MR-Egger [28] and MR-Pleiotropy Residual Sum and Outlier (MR-PRESSO) [29] methods and modified second weights of radial regression of MR (RadialMR) [30]. The intercept of MR-Egger served as a test to assess overall unbalanced horizontal pleiotropy. Heterogeneity of the MR results was assessed using Cochran Q-test, and a significance level of *p* < 0.05 indicated the presence of significant heterogeneity. Radial regression of MR (RadialMR) was employed to identify and exclude outliers that potentially had pleiotropic effects. For the instrument variables (IV), we selected genome-wide significant SNPs (*p* < 5 × 10^−8^) from GWAS summary statistics (*p* < 5 × 10^−6^ for PER due to too less SNPs) and clumped SNPs with an r^2^ < 0.001 within a 10,000 kb range. After harmonization, palindromic and incompatible SNPs were excluded. F-statistics and R^2^ were used to evaluate the strength of the selected SNPs. Additionally, single-SNP and leave-one-out analyses were conducted to investigate whether any individual SNP had a considerable influence on the IVW point estimate [31, 32]. A threshold of *p* < 0.004 (0.05/12) and *p* < 0.05 were used to represent statistical significance and suggestive significance respectively.

#### Multivariable MR (MVMR) analysis

BMI has been reported to have a causal association with PER [33]. The GWAS summary statistics for BMI was obtained from the GIANT consortium without any UKBioBank participants [34]. For the significant causal association (*p* < 0.05) in the univariable MR analysis for glycemic traits or T2D on the risk of PER, the MVMR was performed using the MVMR-IVW and MVMR-MR-Egger method aiming to adjust for potential confounding factor BMI. MVMR-IVW with multiplicative random effects will be used if heterogeneity (*p* < 0.05) is present.

## Results

### Overall genetic correlation

After adjusting for multiple testing (*p* < 0.05/12), we found a substantial positive genetic correlation between PER and T2D (r_g_ = 0.235, *p* = 5.317 × 10^−9^), as well as T2DadjBMI (r_g_ = 0.130, *p* = 3.192 × 10^−3^), which decreased by almost half using LDSC. (Supplementary Table 2). Regarding glycemic traits, HbA1c showed a strong significant positive correlation with PER (r_g_ = 0.229, *p* = 1.766 × 10^−6^). However, no significant overall genetic correlation was observed with other glycemic traits (FG (r_g_ = −0.068, *p* = 0.214), FI (r_g_ = −0.180, *p* = 0.019), HOMA-β (r_g_ = −0.019, *p* = 0.853) and HOMA-IR (r_g_ = 0.091, *p* = 0.450)). No significant overall genetic correlations were observed between glycemic traits (adjusted for BMI) and PER. (FGadjBMI (r_g_ = 0.044, *p* = 0.383), FIadjBMI (r_g_ = 0.019, *p* = 0.765), GladjBMI (r_g_ = −0.002, *p* = 0.974), HOMA-βadjBMI (r_g_ = −0.060, *p* = 0.515) and HOMA-IRadjBMI (r_g_ = −0.048, *p* = 0.611)).

In GNOVA, we also found a strong positive overall genetic correlation between T2D (r_g_ = 0.261, *p* = 2.65 × 10^−13^) and T2DadjBMI (r_g_ = 0.158, *p* = 2.34 × 10^−6^) (Supplementary Table 2). For glycemic traits, HbA1c showed a significant strong genetic correlation with PER (r_g_ = 0.1817, *p* = 4.14 × 10^−6^) and other glycemic traits: FG (r_g_ = −0.031, *p* = 0.565), FI (r_g_ = −0.155, *p* = 0.049), HOMA-β (r_g_ = 0.058, *p* = 0.457) and HOMA-IR (r_g_ = 0.171, *p* = 0.039) did not observe any significant overall genetic correlation, while we did not observe any significant overall genetic correlation with other glycemic traits adjusted for BMI: FGadjBMI (r_g_ = −0.025, *p* = 0.503), FIadjBMI (r_g_ = 0.035, *p* = 0.479), GladjBMI (r_g_ = −0.058, *p* = 0.284), HOMA-βadjBMI (r_g_ = −0.086, *p* = 0.161) and HOMA-IRadjBMI (r_g_ = −0.147, *p* = 0.048).

### Local genetic correlation

For glycemic traits, we found four significant genomic regions between FGadjBMI and PER, with three showing positive local genetic correlation (Supplementary Table 4). One significant genomic region was identified between GladjBMI and PER, also with positive local genetic correlation. Additionally, we discovered four significant genomic regions between HbA1c and PER, all showing positive local genetic correlation. Two significant genomic regions were found between FG and PER, and one significant genomic region was found between HOMA-β and PER. In T2D, we detected five significant genomic regions associated with PER, three of which exhibited positive local genetic correlation. Furthermore, in T2DadjBMI, we found four significant genomic regions related to PER, with three showing negative local genetic correlation. Three genomic regions (chr1:39537291-40933221, chr4:103388441-104802530, and chr5:101101325-102681586) were shared between T2D independent of BMI. We also identified a shared genomic region (chr3:170159134-171311936) between PER and both T2DadjBMI and FGadjBMI. Moreover, there was an overlapped genomic region (chr17:46828412-48027295) among T2D, GladjBMI, and HbA1c.

### Cross-trait meta-analysis

There were 62 independent pleiotropic SNPs identified between PER and glycemic traits or T2D (Supplementary Table 3). Furthermore, none of these 62 SNPs had previously been associated with PER at a threshold of genome-wide significance, while many of them (44 out of 62) had been associated with at least one glycemic trait or T2D.

A total of 32 SNPs were found to be common between T2D and PER. The location that was most prominently shared between two conditions, T2D and T2DadjBMI-PER, was found near *TH*. This location was identified by the presence of a specific genetic variant, rs4929965 (p_CPASSOC_ = 3.57 × 10^−27^) in T2D. The same location was also shared by T2DadjBMI-PER, where the lead genetic variant was rs7482891 (p_CPASSOC_=4.34 × 10^−22^). The second strongest signal was rs5398, located near *SLC2A2* (p_CPASSOC_ = 2.79 × 10^−23^). Finally, the third strongest signal was rs6971365 (p_CPASSOC_ = 1.40 × 10^−18^), located near *LOC10537508*, which was also shared by T2DadjBMI-PER, where the lead SNP was rs697135 (p_CPASSOC_ = 1.37 × 10^−18^). In addition, 12 independent SNPs were shared between T2DadjBMI and PER. The most significant SNP was the same as T2D located near *TH*. For FGadjBMI and PER, the most significant shared locus was near *G6PC2*, which was also shared by HbA1c-PER, HOMA-βadjBMI-PER and FG-PER. For HOMA-β and PER, two SNPs (rs560887 and rs1402837) were shared which rs1402837 located near *G6PC2* was also shared by HOMA-βadjBMI. Two SNPs were shared between FIadjBMI and PER (rs6855363 and rs6108030). One SNP was shared between HOMA-IRadjBMI and PER, which was also shared by T2DadjBMI-PER. Five SNPs were shared between HbA1c and PER. No significant SNP was found between FI, HOMA-IR, GladjBMI and PER.

### Pairwise colocalization analysis

There were eight significant genomic regions with PPA4 > 0.5 between T2D or glycemic traits and PER, and three regions (T2DadjBMI: chr12:83502773-84303690 and chr19:51533417-52984982, HbA1c: chr19:51533417-52984982) with PPA4 > 0.9 (Supplementary Table 5). In addition, sixteen genomic regions with PPA3 > 0.5 were identified between T2D or glycemic traits and PER (Supplementary Table 5). There were nine genomic regions with PPA3 > 0.8 (T2D: chr12:83502773-84303690 and chr12:84303844-85990038, FG: chr20:39611802-40585492, HOMA-β: chr2:167355970-169967174, chr10:677550128:69896336 and chr11:92077144-93273729, FGadjBMI: chr15:73628714-76398392, HOMA-βadjBMI: chr2:167355970-169967174 and chr4:157486535-158742582), and two of the genomic regions (chr12:83502773-84303690 and chr12:84303844-85990038) were mediated by BMI in T2D. The genomic regions for FGadjBMI (chr15:73628714-76398392 and rs12905199) and HOMA-βadjBMI (chr2:167355970-169967174 and rs537183) (PPA3 > 0.5) were also consistent with the CPASSOC results.

### Pathway enrichment analysis

Nine significant pathways were identified between T2D or glycemic trait and PER after multiple correction testing (Supplementary Table 6). For T2D, three pathways were identified, with the response to the glucose pathway being the most significant. For T2DadjBMI, two pathways were identified, with the N6-threonylcarbomyladenosine methylthiotransferase activity pathway being the most significant. For HOMA-βadjBMI, three pathways were identified with the canalicular bile acid transmembrane transporter activity being the most significant. For FG, Ndc80 complex pathway was identified. There were no significant enriched pathways for genes overlapping PER and/or HbA1c, FI, HOMA-β, HOMA-IR, FGadjBMI, FIadjBMI, GladjBMI and HOMA-IRadjBMI.

### Transcriptome-wide association studies

After considering multiple testing correction (Supplementary Fig. 1), the single-trait TWAS method detected 36 genes that have a significant association with PER. For T2D, a total of 5689 genes were found to be significantly associated, while 3197 genes were associated with T2DadjBMI, 2405 genes with HbA1c, 627 genes with FG, 152 genes with FI, 16 genes with HOMA-β, 6 genes with HOMA-IR, 584 genes with FIadjBMI, 1402 genes with FGadjBMI, 91 genes with GladjBMI, 60 genes with HOMA-IRadjBMI, and 83 genes with HOMA-βadjBMI. However, we did not find any genes that were common between PER and glycemic traits or T2D.

### Bidirectional Mendelian Randomization analysis

The genetic liability of PER was suggestively casually associated with lower FG (beta: −0.084, [95%CI −0.166 to –0.001; *p* = 0.048]) and FI (beta: −0.084, [95%CI −0.166 to –0.001; *p* = 0.048]) level. However, the effects disappear after adjusted for BMI. The genetic liability of PER was not causally associated with other glycemic traits and T2D (unadjusted and adjusted for BMI) (Table 1).

**Table 1:** Associations of genetically predicted periodontal disease with Type 2 Diabetes and glycemic traits risks in MR analyses

Exposure	Outcome	Beta or Odd Ratio (IVW)	SE	*P*-value
Periodontal disease	Type 2 Diabetes	1.108	0.073	0.159
	Fasting Glucose	−0.084	0.042	0.048
	Fasting Insulin	−0.084	0.042	0.048
	HOMA-β	0.036	0.068	0.599
	HOMA-IR	−0.036	0.078	0.641
	HbA1c	0.023	0.020	0.255
	Type 2 Diabetes adjusted for body mass index	1.023	0.096	0.816
	Fasting Glucose adjusted for body mass index	0.012	0.025	0.632
	Fasting Insulin adjusted for body mass index	0.019	0.024	0.410
	2 h post oral glucose adjusted for body mass index	0.006	0.022	0.780
	HOMA-β adjusted for body mass index	−0.045	0.054	0.398
	HOMA-IR adjusted for body mass index	−0.035	0.051	0.489

*MR* Mendelian randomization, *HbA1c* glycated hemoglobin, *HOMA-IR* Homeostasis Model Assessment of Insulin Resistance, *HOMA-β* Homeostasis Model Assessment of Beta-cell function, *IVW* Inverse variance weighted, *SE* Standard error

Significant causal effect of genetically predisposed HOMA-β (OR: 0.823 [95%CI 0.697–0.950; *p* = 2.63 × 10^−3^]) and FG (OR: 0.943 [95%CI 0.905–0.982; *p* = 3.18 × 10^−3^]) (Table 2) on PER were observed using the IVW approach. The estimates remain directionally consistent in all approaches except the simple median method. However, leave one out analysis revealed that rs560887 in *G6PC2* and rs853777 in *ABCB11* as the SNP causing the casual effect for HOMA-β and FG respectively (Supplementary Fig. 3). In addition, a suggestive casual effect of genetically predisposed T2D (OR: 1.016 [95%CI 1.005–1.027; *p* = 6.24 × 10^−3^]) and HbA1c (OR: 1.124 [95%CI 1.039–1.208; *p* = 7.00 × 10^−3^]) on PER were observed using the IVW approach while other glycemic traits showed no causal associations between PER. The estimates remain directionally consistent in IVW radial and MR-PRESSO approach. When the effect of BMI was removed, only HOMA-βadjBMI (OR: 0.762 [95%CI 0.631–0.893; *p* = 4.55 × 10^−5^]) showed significant associations with lower risk of PER.

**Table 2:** Associations of genetically predicted Type 2 Diabetes and glycemic traits with periodontal diseases risks in MR analyses

Exposure	Outcome	Odd Ratio (IVW)	SE	*P*-value
Type 2 Diabetes	Periodontal disease	1.012	0.006	0.006
Fasting Glucose		0.943	0.020	0.003
Fasting Insulin		0.964	0.068	0.590
HOMA-β		0.823	0.065	0.003
HOMA-IR		1.058	0.076	0.456
HbA1c		1.124	0.117	0.007
Type 2 Diabetes adjusted for body mass index		1.002	0.005	0.698
Fasting Glucose adjusted for body mass index		1.005	0.034	0.887
Fasting Insulin adjusted for body mass index		1.002	0.049	0.961
2 h post oral glucose adjusted for body mass index		1.012	0.019	0.530
HOMA-β adjusted for body mass index		0.762	0.067	4.55 × 10^−5^
HOMA-IR adjusted for body mass index		0.939	0.085	0.460

*MR* Mendelian randomization, *HbA1c* Glycated hemoglobin, *HOMA-IR* Homeostasis Model Assessment of Insulin Resistance, *HOMA-β* Homeostasis Model Assessment of Beta-cell function, *IVW* Inverse variance weighted, *SE* Standard error

### MVMR analysis

In the MVMR-IVW analysis, the causal association between HOMA-β and PER was similar (OR: 0.725 [95% CI: 0.619–0.847; *p* < 0.001]), with conditional F-statistics 3.6 (less than 10), but the effect was confirmed by consistent MVMR-MR-Egger method (Supplementary Table 11). However, the genetic liability of T2D and FG does not affect the risk of PER after adjusted for BMI with conditional F-statistics exceeding 10.

## Discussion

As far as we know, this is the first comprehensive genome-wide study that examines the genetic relationship, pleiotropic regions, and expression-trait associations between PER and T2D or glycemic traits (unadjusted and adjusted for BMI). Our findings show a positive global genetic correlation between T2D-PER, T2DadjBMI-PER, and HbA1c-PER, indicating a shared genetic basis. Pairwise colocalization analysis also validated some pleiotropic SNPs between PER and T2D or glycemic traits (unadjusted and adjusted for BMI). Pathway enrichment analysis showed that several pathways like bile acid secretion and response to glucose were linked between T2D or glycemic traits (unadjusted and adjusted for BMI) and PER. We also discovered a significant causal relationship between genetically predicted FG, HOMA-β, and HOMA-βadjBMI for PER, with non-significant associations when excluding specific SNPs (rs507666 in HOMA-β and rs853777 in FG).

Our research aligns with previous studies but expands on these findings in significant ways. Firstly, by utilizing summary data from previous GWAS, we significantly increased the statistical power of genetic correlation analysis. Shungin et al. previously reported a positive genetic correlation between T2D and PER (*p* < 0.05) in a T2D GWAS with 12,171 cases and 56,682 controls. In our study, with a sample size ten times larger, we also found a significant positive genetic correlation between T2D, T2DadjBMI, and PER. While a previous study by the same author did not find a significant genetic correlation between HbA1c and PER in 46,368 individuals, our analysis, with a sample size four times larger, supports the finding of a significant positive genetic correlation [14]. The second important aspect of our study is the consideration of the effect of BMI. Previous genetic studies have suggested that the association between T2D or glycemic traits and PER could be entirely explained by BMI. Our research shows that there is a pathogenesis pathway that is independent of BMI. Our study is the first to examine the overall and local genetic correlation between T2D or glycemic traits that have been adjusted for BMI and PER. Our bidirectional MR analysis showed that FG, HOMA-β and HOMA-βadjBMI were significantly associated with PER, while T2D and HbA1c were suggestively associated with PER. Previous MR analyses reported a null causal association between T2D and PER [35, 36]. However, we discovered a novel T2D-PER and HOMA-βadjBMI-PER association (univariable MR and multivariable MR). Removing the effect of BMI resulted in no causal relationship between T2D-PER and FG-PER. Our findings for HbA1c-PER aligned with previous studies [35, 36].

Our pairwise colocalization and CPASSOC analysis also identified pleiotropic SNPs shared between PER and glycemic traits or T2D. We have identified three novel SNPs (rs12905199 for FGadjBMI-PER near *CSK*, rs13108763 for T2DadjBMI-PER near *PDGFC*, and rs560887 for HbA1c-PER near *G6PC2* and *SPC25*). The first of these SNPs, rs12905199, is located near the gene *CSK*, which is shared by FGadjBMI and PER, and overlaps with FGadjBMI-PER in GWAS-PW with PPA3 > 0.8. *CSK* regulates SRC family kinases, playing a role in T-cell activation and osteoclast activity [37]. Altered osteoclast activity is seen in hyperglycemia and periodontitis [38, 39]. *CBL*, a SRC family kinase, inhibits *NLRP3* inflammasome activation by downregulating glucose transporter 1 [40]. Individuals with chronic or aggressive periodontitis show elevated *NLRP3* expression levels in gingival tissue compared to healthy controls [41]. A study found that patients with PER and PER + T2D had higher serum and salivary *NLRP3* concentrations compared to T2D patients and normal controls [42]. The expression of *NLRP3* was influenced by patients with T2D, regardless of their BMI status [43]. Hyperactive SRC family kinases impair glucose metabolism in pancreatic beta cells, potentially raising fasting glucose levels [44]. The second novel SNP is rs13108763 near *PDGF-C*, a gene that encodes a protein in the PDGF family and common to T2D, T2DadjBMI and HOMA-IRadjBMI. PDGF acts as a chemoattractant for neutrophils, monocytes/macrophages, and fibroblasts, while also stimulating the proliferation of mesenchymal cells such as periodontal ligament cells [45, 46]. *PDGFC* has a critical role in angiogenesis, and patients with PER often have abnormal angiogenesis that leads to progressive inflammation, which is a sign of the condition [47]. Increased liver methylation expression in *PDGFA* is associated with hyperinsulinemia and insulin resistance, even after adjusting for BMI and other factors [48]. *PDGFC* is potentially involved in periapical lesion pathogenesis through inflammation and contributes to complications in type 2 diabetes, including diabetic foot, microangiopathy, and nephropathy [49, 50]. Previous study showed that the third novel SNP rs560887, shared by HOMA-βadjBMI, FGadjBMI, and HbA1c, is located near *G6PC2* and *SPC25*. *G6PC2* encodes an enzyme involved in releasing glucose into the bloodstream and is associated with lower fasting plasma glucose levels in mice [51]. *G6PC2* in pancreatic beta cells affects the threshold for glucose-induced insulin secretion, impacting FG levels [52]. Variants in *SPC25* also showed an association with tooth morbidity but the biological mechanism remains unknown [53]. A recent study demonstrated that this SNP reached genome-wide significance and was associated with reduced HbA1c levels after adjusting for BMI [54]. These discoveries indicate that there is a biological mechanism at play that is not related to BMI.

It is important to recognize some limitations of our study. Firstly, we focused on populations of European ancestry, which limits the generalizability of our findings to non-European individuals. Secondly, we were unable to conduct sex specific GWAS analyses on T2D and glycemic traits due to limited data availability. This meant that we were not able to match the exposure data on PER with sex-specific data, even though males are more susceptible to the risk and severity of PER and T2D [55, 56]. Thirdly, the PER GWAS summary statistics were obtained through a meta-analysis of GLIDE and UKB datasets. It is important to acknowledge that the UKB data relied on self-reported oral health information, which introduces the possibility of misdiagnosis or inaccuracies. Further cross-sectional, longitudinal, and experimental studies, including animal experiments, are needed to elucidate the biological mechanisms underlying the observed genetic correlation. Fourthly, limited by small sample sizes, the available GWAS summary statistics for HOMA-β and HOMA-IR (unadjusted and adjusted for BMI) lack the power to establish a definitive pleiotropic connection between PER and these traits independent of BMI. More substantial GWAS summary statistics are required for conclusive results. Replication of our findings using publicly accessible T2DadjBMI GWAS datasets is currently not feasible. Additionally, the power for the results of T2D on PER in the MR analysis is comparatively small (31%) due to smaller effect sizes observed in the current MR study compared to previous observational studies [57]. Lastly, both GWAS summary statistics for T2D and PER involve UKB participants which can lead to overfitting bias in MR, but bias due to sample overlap is minimal when the instruments have sufficient strength (F-statistics > 10) [58, 59]. In addition, with relatively large sample sizes, the bias due to sample overlap is expected to be very small [60].

To summarize, our study utilized the most extensive GWAS summary statistics and conducted a genetic correlation analysis, which revealed a robust association between PER and T2D or glycemic traits. The causal association remained significant for HOMA-β after controlling BMI. These findings offer valuable new insights into the observed connections between these conditions at a molecular level. Our research demonstrates that the genetic predisposition to T2D or glycemic traits have pleiotropic effects on the development of PER, independent of BMI, as shown by the statistical significance of CPASSOC. This reinforces the notion that there are shared biological processes between these conditions.

### Supplementary information

Supplementary figures

Supplementary tables
