# Supplementary figures and images for: Shared genetic architecture between periodontal disease and type 2 diabetes: a large scale genome-wide cross-trait analysis
Source: Endocrine. 2024 Mar 9;85(2):685–94. doi: 10.1007/s12020-024-03766-8 (PMC11291565; doi:10.1007/s12020-024-03766-8)

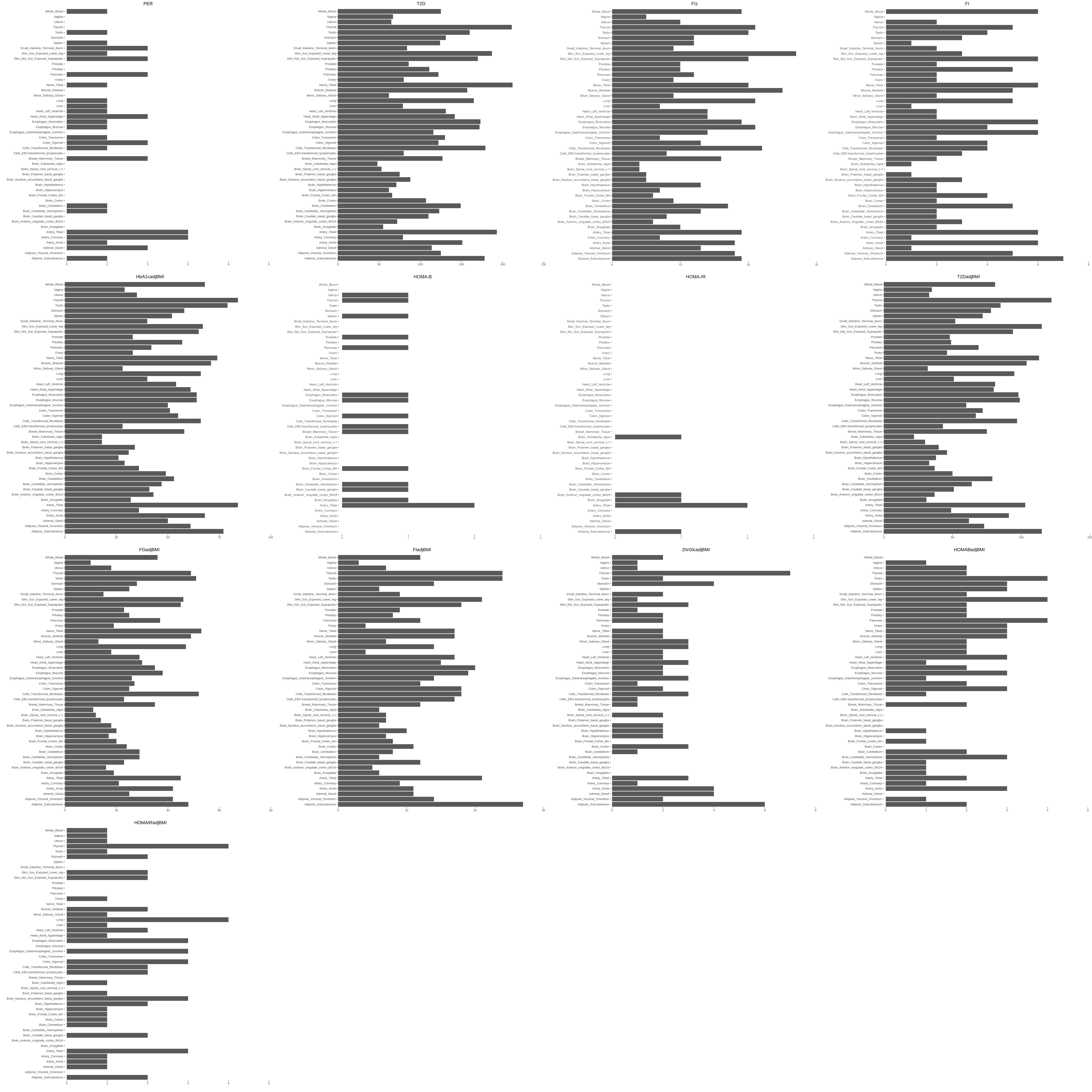

Supplement: Supplementary file 1 — Supplementary figures [file 12020_2024_3766_MOESM1_ESM.zip › perglu_suppfig1_revised.jpg]

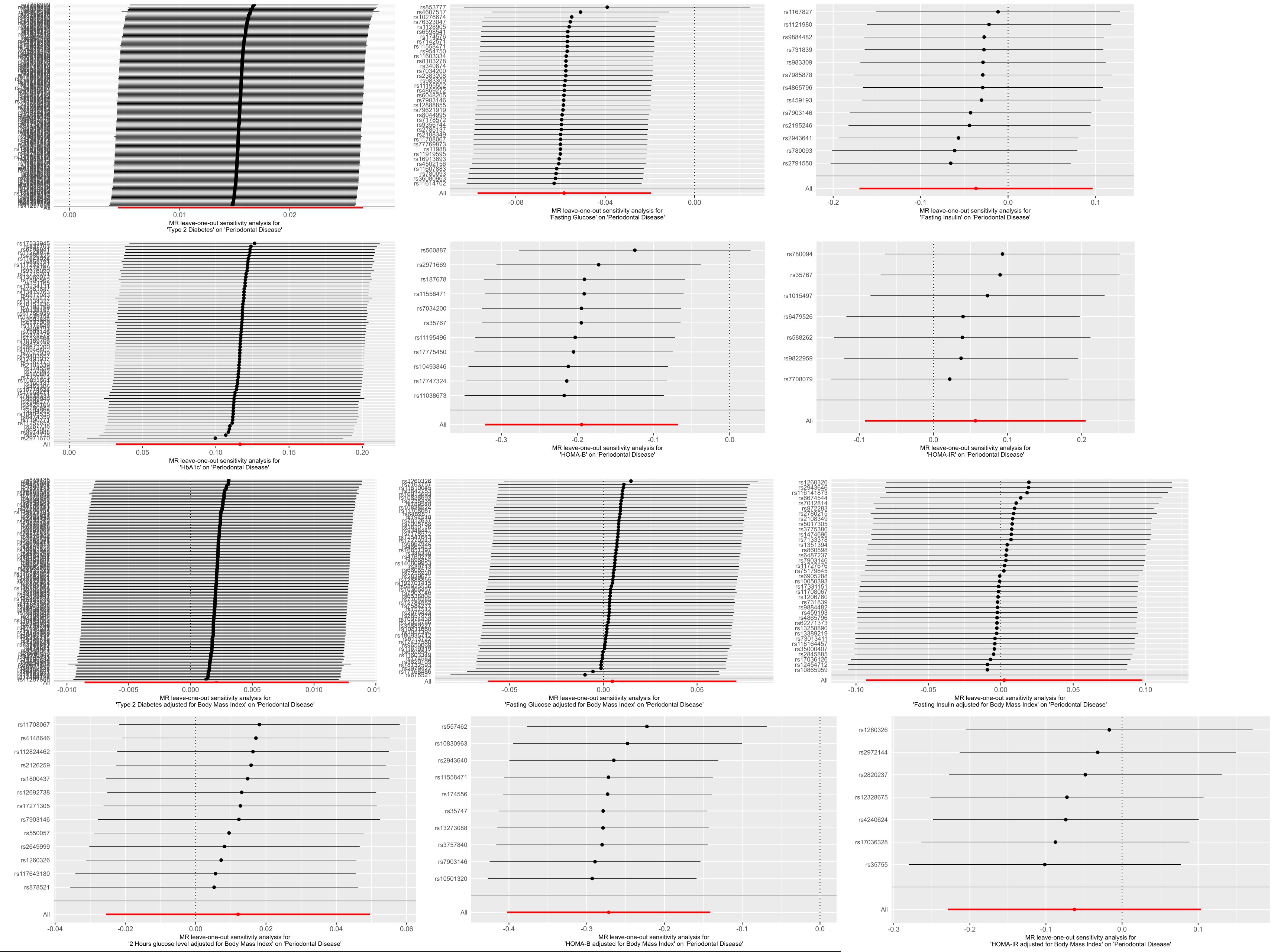

Supplement: Supplementary file 1 — Supplementary figures [file 12020_2024_3766_MOESM1_ESM.zip › pergly_suppfig3_revsied.jpg]
